# The Arginine Biosynthesis Pathway of Candida albicans Regulates Its Cross-Kingdom Interaction with Actinomyces viscosus to Promote Root Caries

**DOI:** 10.1128/spectrum.00782-22

**Published:** 2022-07-13

**Authors:** Kaixin Xiong, Hualing Zhu, Yanyao Li, Mengzhen Ji, Yujia Yan, Xuan Chen, Yaqi Chi, Xueqin Yang, Ling Deng, Xuedong Zhou, Ling Zou, Biao Ren

**Affiliations:** a State Key Laboratory of Oral Diseases, National Clinical Research Center for Oral Diseases, West China School of Stomatology, Sichuan University, Chengdu, China; b State Key Laboratory of Oral Diseases, National Clinical Research Center for Oral Diseases, Department of Conservation Dentistry and Endodontics, West China School of Stomatology, Sichuan University, Chengdu, China; University of Michigan

**Keywords:** root caries, multispecies infection, Candida *albicans*, *Actinomyces viscosus*, arginine biosynthesis pathway

## Abstract

The cross-kingdom interactions between Candida albicans and Actinomyces viscosus play critical roles in root caries. However, the key pathway by which C. albicans regulates its interactions with A. viscosus is unclear. Here, we first employed 39 volunteers with root caries and 37 caries-free volunteers, and found that the abundances of C. albicans and A. viscosus were significantly increased in the individuals with root caries and showed a strong positive correlation. Their dual-species combination synergistically promoted biofilm formation and root caries in rats. The arginine biosynthesis pathway of C. albicans was significantly upregulated in dual-species biofilms and dental plaques from another 10 root caries volunteers compared with the 10 caries-free volunteers. The exogenous addition of arginine increased the cariogenicity of the dual-species biofilm. The C. albicans *ARG4*, a key gene from the arginine biosynthesis pathway, null mutant failed to promote dual-species biofilm formation and root caries in rats; however, the addition of arginine restored its synergistic actions with A. viscosus. Our results identified the critical roles of the C. albicans arginine biosynthesis pathway in its cross-kingdom interactions with A. viscosus for the first time and indicated that targeting this pathway was a practical way to treat root caries caused by multiple species.

**IMPORTANCE** Root caries is a critical problem that threatens the oral health of the elderly population. Our results identified the essential roles of the C. albicans arginine biosynthesis pathway in its cross-kingdom interactions with A. viscosus in root caries for the first time and indicated that targeting this pathway was a practical way to treat root caries caused by multiple species.

## INTRODUCTION

Oral diseases are a major health burden for many countries and affect individuals throughout their lifetimes, causing pain, discomfort, disfigurement, and even death (https://www.who.int/news-room/fact-sheets/detail/oral-health). Oral diseases affect approximately 3.5 billion people worldwide, of which 2.3 billion people suffer from permanent tooth caries, the most common oral problem (1). Root caries is among the important reasons for tooth loosening in the elderly population, with prevalence ranging from 25% to 100% (2–4). Facilitating the prevention and treatment of root caries is among the important missions necessary to achieve the plan “8020 better oral health for older people” proposed by the WHO (5).

Oral microorganisms have been indicated to play crucial roles in the development of root caries. Actinomyces viscosus is an early colonizing microorganism of the root surface and a key pathogenic agent for root caries. A. viscosus was found to be the dominant bacterium in all plaque samples from root surface caries (6) and accounted for 100% of the isolation frequencies (7). The cariogenic factors of A. viscosus include the strong ability of cell adhesion (8) and the capability to metabolize several kinds of carbohydrates, such as starch, sucrose, glucose, and fructose, which results in the production of large amounts of acids and the rapid demineralization of the infected teeth (9). Acid production can also reduce the growth of other oral bacteria in root caries plaque because A. viscosus has a tolerance to acid (9).

Candida albicans is a common symbiotic fungus in the oral cavity, respiratory and digestive tracts, and urogenital system. C. albicans is highly associated with oral candidiasis (10) and dental caries in the oral cavity, especially in Early Childhood Caries (ECC) and root caries (11, 12). The isolation frequency of C. albicans in root caries lesions was found to be approximately 40% (13), while both the isolation frequency and detection abundance of C. albicans from root caries lesions were found to be much higher than those from sound root surfaces (12). C. albicans can penetrate into the dentin tubules and bind to collagen, and then secrete hydrolases to degrade collagen under acidic conditions to promote the caries process (14, 15).

The cross-kingdom interactions between C. albicans and many oral bacteria, such as Streptococcus, *Actinomyces*, *Fusobacterium*, and *Helicobacter* species, contribute to the development of different oral diseases (16–19). C. albicans and *Actinomyces* could coaggregate tightly, especially when C. albicans was in the hyphal state (20, 21). C. albicans and *streptococci* had a synergistic partnership, as *streptococci* promoted C. albicans to invade the oral mucosae, while C. albicans promoted *streptococci* to form biofilms on abiotic surfaces and in the oral cavity (18, 22, 23). Interactions between Streptococcus mutans and C. albicans could result in the formation of a more complicated biofilm with the elevation of extracellular polysaccharide production by S. mutans and hyphal formation of C. albicans, respectively, to promote dental caries development (16, 24–26). We also found that C. albicans could affect the interactions between S. mutans and Streptococcus sanguinis to promote the development of dental caries (12). Streptococcus gordonii could also promote C. albicans biofilm formation and hyphal development (17). C. albicans and Staphylococcus aureus synergistically interacted to promote pathogenic potential, increase resistance to antibiotics and help *Candida* circumvent the host immune system (27, 28). However, the key pathways by which C. albicans regulates its interactions with oral bacteria are still unclear.

We previously found that C. albicans increased the cariogenic abilities of A. viscosus *in vitro,* while voriconazole inhibited their cross-kingdom interactions (29, 30). However, the key pathway by which C. albicans regulates its cross-kingdom interactions with A. viscosus and the effects of their coinfection in root caries are still unclear. Thus, in this study, we aimed to identify the C. albicans pathway that is critical for its interactions with A. viscosus in clinical root caries samples, dual-species biofilms, and root caries rat models.

## RESULTS

### Increased detection rates and abundances of C. albicans and A. viscosus in root caries.

**FIG 1:**
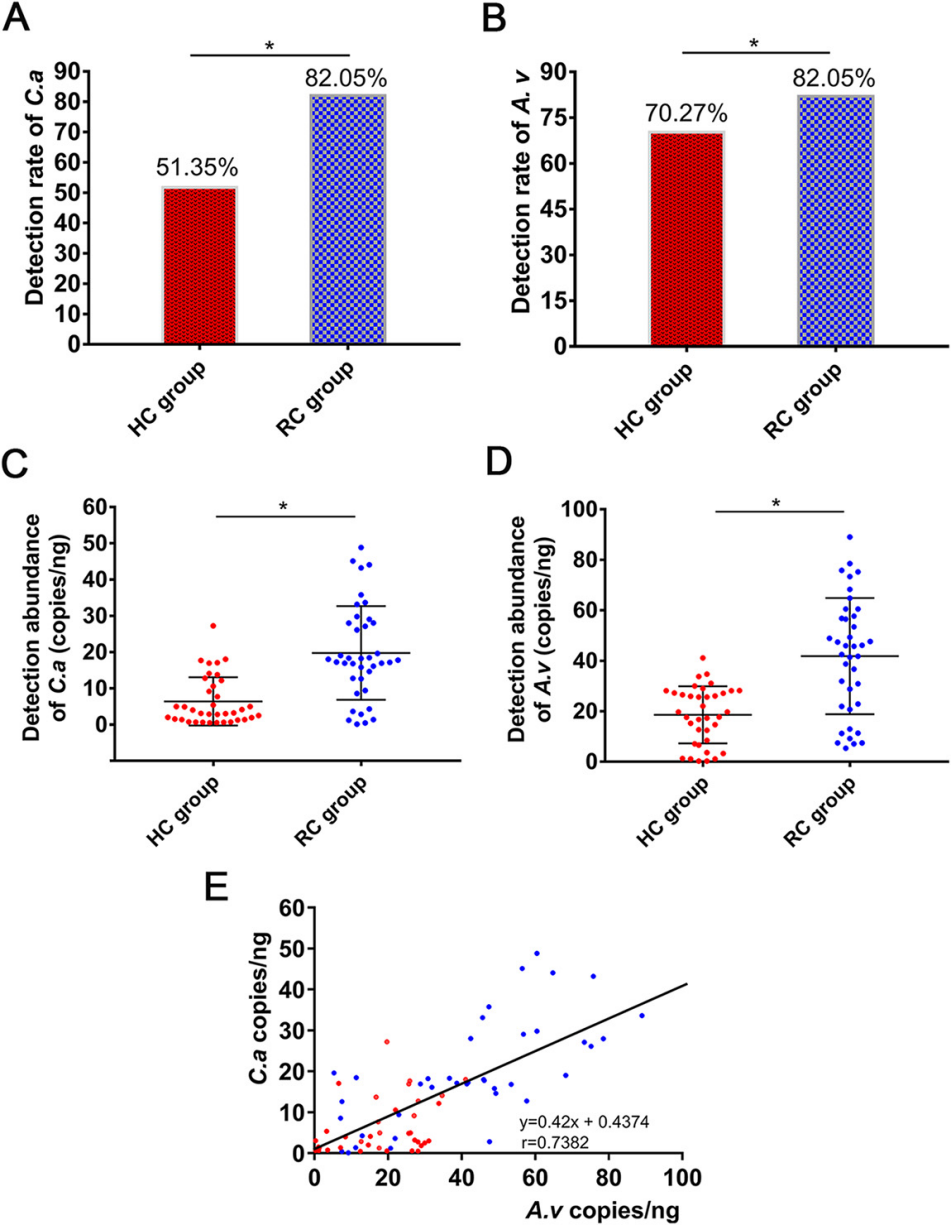
Increased amounts of C. albicans and A. viscosus in root carious lesions. (A, B) Detection rates of C. albicans and A. viscosus by PCR in the RC and HC groups. (A) C. albicans; (B) A. viscosus. (*, *P* < 0.05). (C, D) The abundances of C. albicans and A. viscosus determined by qPCR in the RC and HC groups. (C) C. albicans; (D) A. viscosus (*, *P* < 0.05). (E) Correlation and linear regression analysis between the abundances of C. albicans and A. viscosus among the plaque samples of all recruited subjects. The red dots represented samples from the HC group and the blue dots represented samples from the RC group. The Pearson correlation coefficient *r* value is 0.7382 (*r* = 0.7382, r2 = 0.5449, *P* ≤ 0.05).

Seventy-six volunteers, including 39 patients with root caries in the root caries (RC) group and 37 healthy people in the healthy control (HC) group, were recruited to compare the detection rates and abundances of C. albicans and A. viscosus in supragingival dental plaque. The rate of C. albicans detection was 82.05% in the RC group and was significantly higher than that in the HC group (51.35%) (Fig. 1A; *P* < 0.05). The rate of A. viscosus detection was 82.05% in the RC group and was also significantly higher than that in the HC group (70.27%) (Fig. 1B; *P* < 0.05). The abundances of C. albicans and A. viscosus were also significantly enriched in the RC group (19.75 ± 12.92 copies/ng and 41.84 ± 23 copies/ng, respectively) compared with those in the HC group (6.399 ± 6.669 copies/ng and 18.57 ± 11.33 copies/ng, respectively) (Fig. 1C and D; *P* < 0.05). Notably, the abundances of C. albicans and A. viscosus were positive (Fig. 1E), suggesting that there is a strong correlation between C. albicans and A. viscosus in root caries.

### Synergistic interactions between C. albicans and A. viscosus promoted biofilm formation and cariogenicity.

**FIG 2:**
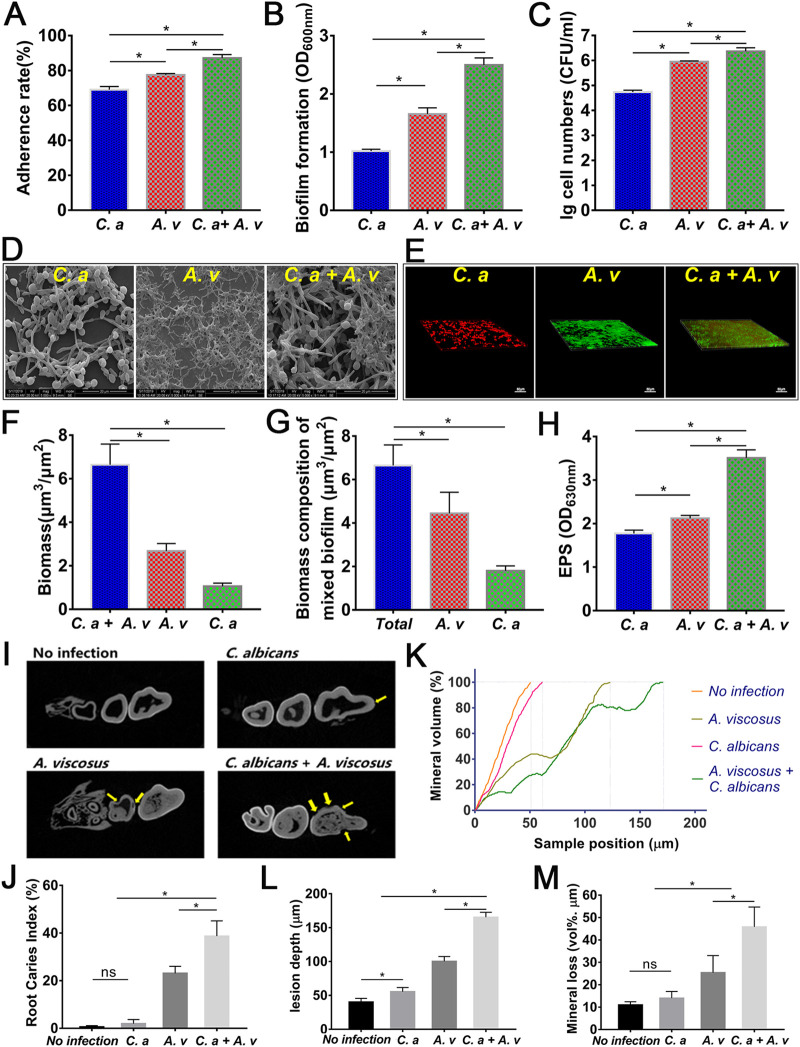
C. albicans synergistically interacted with A. viscosus to promote the cariogenicity and root caries. (A to C) The adherence rates (A), biofilm formations (B), and CFU (C) from the three groups: C. albicans single-species; A. viscosus single species; C. albicans + A. viscosus dual-species (*, *P* < 0.05). (D, E) Structural observations of biofilms formed by C. albicans, A. viscosus, C. albicans, and A. viscosus through SEM (D) and FISH (E) analysis. C. albicans was stained with red color while A. viscosus was stained with green color. (F, G) Biomasses of the three kinds of biofilms from FISH observation result, quantitatively calculated by COMSTAT. (F) Biomasses of C. albicans single-species, A. viscosus single-species, and C. albicans + A. viscosus dual-species FISH-visualized biofilms, respectively, quantified by COMSTAT. (G) Biomass of C. albicans + A. viscosus FISH-visualized biofilm and the respective biomass compositions of C. albicans and A. viscosus in this dual-species biofilm (*, *P* < 0.05). (H) Water insoluble EPS productions of three groups: C. albicans single-species, A. viscosus single species, C. albicans + A. viscosus dual-species (*, *P* < 0.05). (I) Representative micro-CT images of rat jaws from uninfected rats and rats infected with C. albicans, A. viscosus, or C. albicans + A. viscosus, respectively. Yellow arrows indicated root carious lesions. (J) Root caries index scores according to Doff’s system (*, *P* < 0.05; ns, not significant). (K) The mineral volume curves of teeth from uninfected rats and rats infected with C. albicans, A. viscosus, or C. albicans + A. viscosus, respectively. (L) The lesion depths curves of teeth from uninfected rats and rats infected with C. albicans, A. viscosus, or C. albicans + A. viscosus, respectively (*, *P* < 0.05). (M) The mineral losses of teeth from uninfected rats and rats infected with C. albicans, A. viscosus, or C. albicans + A. viscosus, respectively (*, *P* < 0.05; ns, not significant).

We then investigated the cariogenicity of the C. albicans and A. viscosus dual-species combinations due to their positive correlation in clinical samples. During biofilm formation, the adherence rate in the dual-species group (87.17%) was higher than that in the single-species groups (69.06% for C. albicans, 77.51% for A. viscosus) (Fig. 2A; *P* < 0.05). The biofilm biomass and viable cells of the dual-species group were significantly elevated compared with those of the single-species groups (Fig. 2B and C; *P* < 0.05). These results indicated that the combination of C. albicans and A. viscosus enhanced cell adherence, cell growth, and biofilm formation. The dual-species combination formed thicker and denser biofilms (Fig. 2D and E). A higher proportion of hyphal forms of C. albicans was observed in the dual-species biofilms than in the C. albicans mono-species biofilms (Fig. 2D and E). The numbers of both C. albicans and A. viscosus cells were elevated in the dual-species group (Fig. 2F and G). To test whether viable cell-cell contact is necessary for the enhanced biofilm formation, we combined viable cells, heat-killed cells, and cell supernatants of C. albicans and A. viscosus, respectively, and found that only the combination of viable cells significantly enhanced biofilm formation, indicating that cell-cell contact was essential for the interactions between C. albicans and A. viscosus (Fig. S1).

Moreover, the dual-species biofilm produced more water insoluble extracellular polysaccharides (EPS), the key cariogenic virulence factor, compared with that of C. albicans or A. viscosus single-species biofilms (Fig. 2H; *P* < 0.05), indicating that the cross-kingdom interactions between C. albicans and A. viscosus enhanced cariogenicity.

### C. albicans synergized with A. viscosus to promote root caries in rats.

We further evaluated whether the cross-kingdom interactions of C. albicans and A. viscosus could promote the development of root caries in rat model. Rats infected with C. albicans alone formed very little root caries, while the rats infected with A. viscosus formed typical root caries, indicating the strong cariogenic ability of A. viscosus (Fig. 2I and J). Coinfection with C. albicans and A. viscosus synergistically increased the formation and severity of root caries compared to those of C. albicans or A. viscosus single-species infection (Fig. 2I and J). The rats coinfected with C. albicans and A. viscosus had the highest root caries score, lowest mineral volume, and largest lesion depth and mineral loss of the jaw (Fig. 2J to M; *P* < 0.05). These results demonstrated that C. albicans could synergize with A. viscosus to promote root caries *in vivo*.

### The highly activated C. albicans arginine biosynthesis pathway in dual-species biofilms.

**FIG 3:**
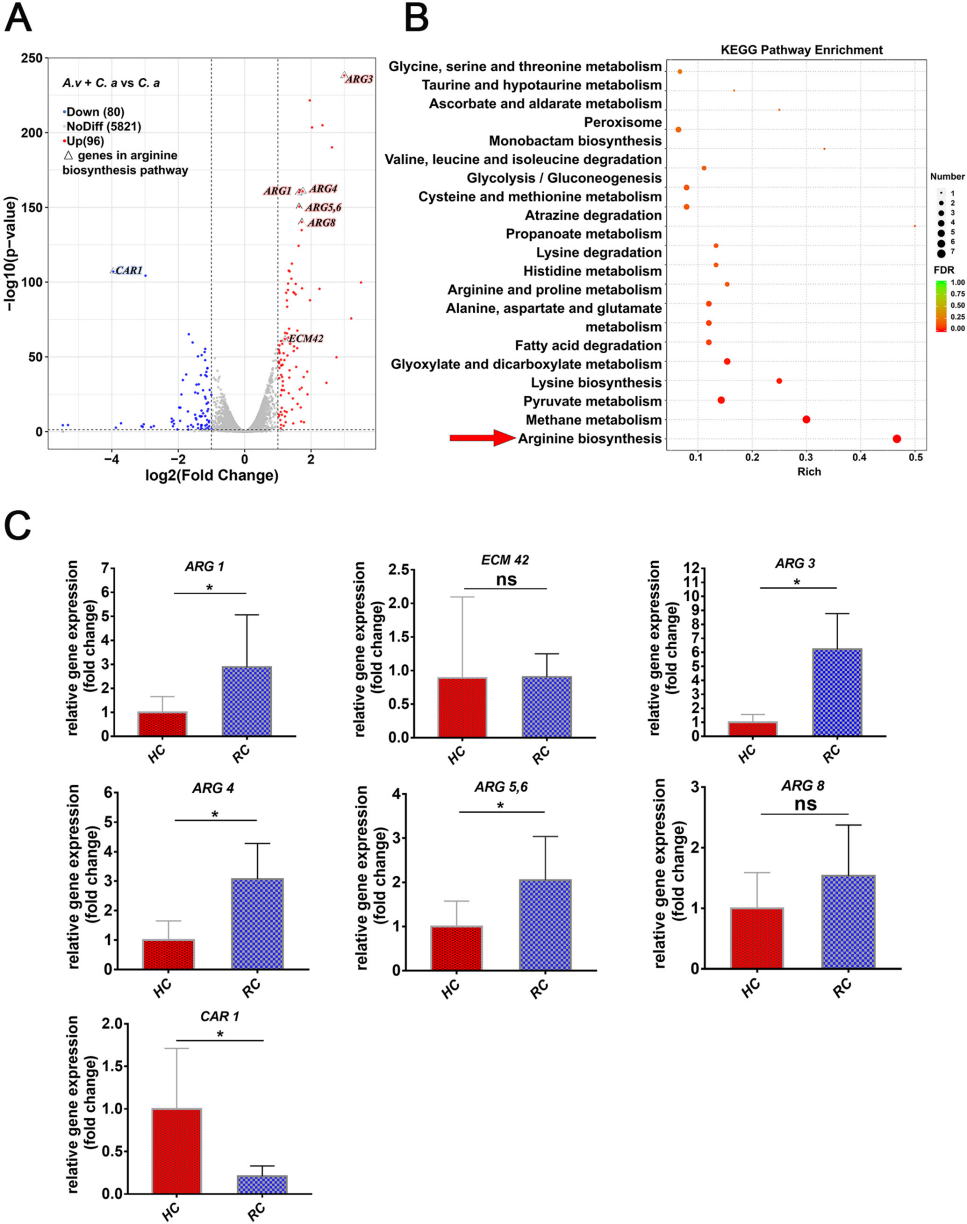
Activation of the C. albicans arginine biosynthesis pathway in dual-species biofilm and clinical root caries plaque samples. (A) Volcano plot of centered and scaled FPKM values of DEGs indicating significant expression changes in the arginine biosynthesis pathway. The *ARG1*, *ECM42*, *ARG3*, *ARG4*, *ARG5*,*6*, *ARG8*, and *CAR1* genes in the arginine biosynthesis pathway were marked. (B) KEGG pathway enrichment analysis indicating that the arginine biosynthesis pathway was the most DEG-enriched pathway. The yellow arrow showed the arginine biosynthesis pathway. (C) The differential expression of the arginine biosynthesis-associated genes: *ARG1*, *ECM42*, *ARG3*, *ARG4*, *ARG5*,*6*, *ARG8*, and the arginine degradation-associated gene: *CAR1* were confirmed from the root caries plaques (RC groups) compared with the HC groups by qPCR (*, *P* < 0.05; ns, not significant).

To further identify the key pathway by which C. albicans regulates its synergistic interaction with A. viscosus, we analyzed the transcriptome of C. albicans from the dual-species biofilm compared with the C. albicans single-species biofilm (Fig. S2). There were 176 differentially expressed genes (DEGs) between the two groups (FDR < 5%, |log2FoldChange| >1). 96 genes were upregulated and 80 genes were downregulated in the dual-species biofilm (Fig. 3A; Fig. S2C). The expressions of genes related to arginine biosynthesis of C. albicans were significantly increased, while the expression of arginine degradation associated gene *CAR1* was significantly decreased (Fig. 3A). Kyoto Encyclopedia of Genes and Genomes (KEGG) analysis further confirmed that the DEGs were most enriched in the arginine biosynthesis pathway of C. albicans (Fig. 3B). Gene expressions in the arginine biosynthesis pathway, including that of *ARG1*, *ECM42*, *ARG3*, *ARG4*, *ARG5*,*6*, *ARG8*, and *CAR1*, were then confirmed by quantitative PCR (qPCR) analysis. The results confirmed that the expression levels of *ARG1*, *ECM42*, *ARG3*, *ARG4*, and *ARG5*,*6* were significantly upregulated and that the expression of *CAR1* was significantly downregulated in the dual-species biofilm compared with those in the C. albicans single-species biofilm (Fig. S3); this result was consistent with the transcriptome analysis (Fig. 3A), indicating the key roles of the arginine biosynthesis pathway in dual-species biofilm.

### Enhanced activation of the C. albicans arginine biosynthesis pathway in clinical root caries.

To further confirm that the arginine biosynthesis pathway of C. albicans was also upregulated in clinical root caries, another 20 volunteers, including 10 patients with root caries (RC group) and 10 caries-free individuals (HC group), were recruited. The expression levels of the genes associated with arginine biosynthesis (*ARG1*, *ARG3*, *ARG4*, and *ARG5*,*6*) were significantly elevated while *CAR1* expression was decreased in the root caries plaques in the RC group compared with those in the sound root surface plaques in the HC group (Fig. 3C), which was in line with the transcriptome analysis in the dual-species biofilms and indicated that the enrichment of the C. albicans arginine biosynthesis pathway played key roles in the development of root caries.

### The addition of arginine increased the growth and biofilm formation of C. albicans, A. viscosus, and their dual-species combination.

**FIG 4:**
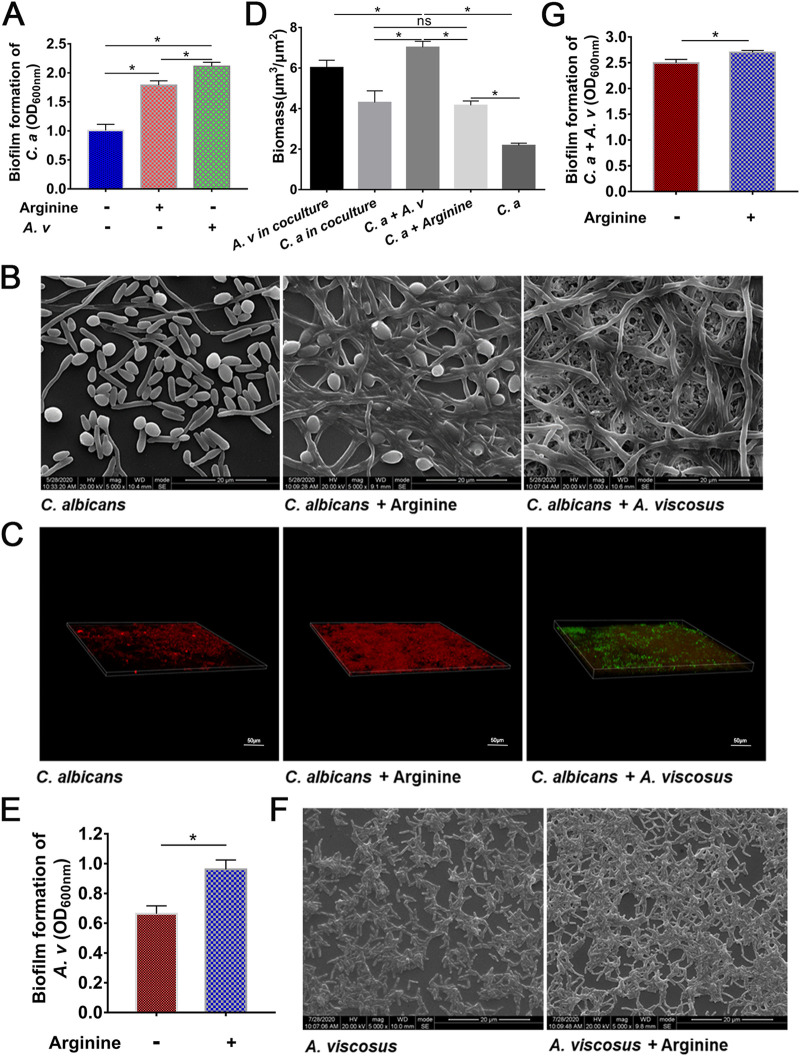
The addition of arginine promoted the growth of C. albicans, A. viscosus, and dual-species biofilms. (A) Effect of arginine on C. albicans biofilm formation: Total biomasses quantified with CV assay of three kinds of biofilms formed by C. albicans, A. viscosus, C. albicans + A. viscosus, respectively (*, *P* < 0.05). (B, C) Effect of arginine on A. viscosus biofilm structure: Three structures of biofilms from C. albicans, A. viscosus, C. albicans + A. viscosus, respectively, observed with SEM and FISH. C. albicans was stained with red color while A. viscosus was stained with green color. (D) Biomasses of the biofilms according to FISH observation quantitatively calculated by COMSTAT (*, *P* < 0.05; ns, not significant). (E) Effect of arginine on the A. viscosus biofilm formation: Total biomasses of A. viscosus biofilm formed with or without arginine addition quantified by CV assay (*, *P* < 0.05). (F) Effect of arginine on A. viscosus biofilm structure: Structures of A. viscosus biofilm formed with or without arginine addition observed by SEM. (G) Effect of arginine on A. viscosus and C. albicans dual-species biofilm formation: Total biomasses of dual-species biofilm formed with or without arginine addition quantified by CV assay (*, *P* < 0.05).

We further evaluated the effect of the corresponding product of the arginine biosynthesis pathway (arginine) on the growth of C. albicans and A. viscosus. As shown in Fig. 4, the addition of arginine promoted C. albicans biofilm formation, similar to the dual-species combination, compared with that in the group without arginine (Fig. 4A; *P* < 0.05). More hyphal formation in the C. albicans single-species biofilm with the addition of arginine and the dual-species biofilm was observed (Fig. 4B). C. albicans formed denser and more compact biofilm with the addition of arginine (Fig. 4C), with higher biomass than that of C. albicans single-species biofilm without arginine (Fig. 4C and D; *P* < 0.05). The addition of arginine also promoted the formation of A. viscosus biofilms. A. viscosus formed denser and more compact biofilms with a greater total biomass with the addition of arginine (Fig. 4E and F). Moreover, the addition of arginine also enhanced the formation of dual-species biofilm. The total biomass of the dual-species biofilm with the addition of arginine was elevated significantly (Fig. 4G; *P* < 0.05), suggesting that arginine increased the growth and biofilm formation of the C. albicans and A. viscosus combination.

### The C. albicans arginine biosynthesis pathway regulated its cross-kingdom interaction with A. viscosus in dual-species biofilms.

**FIG 5:**
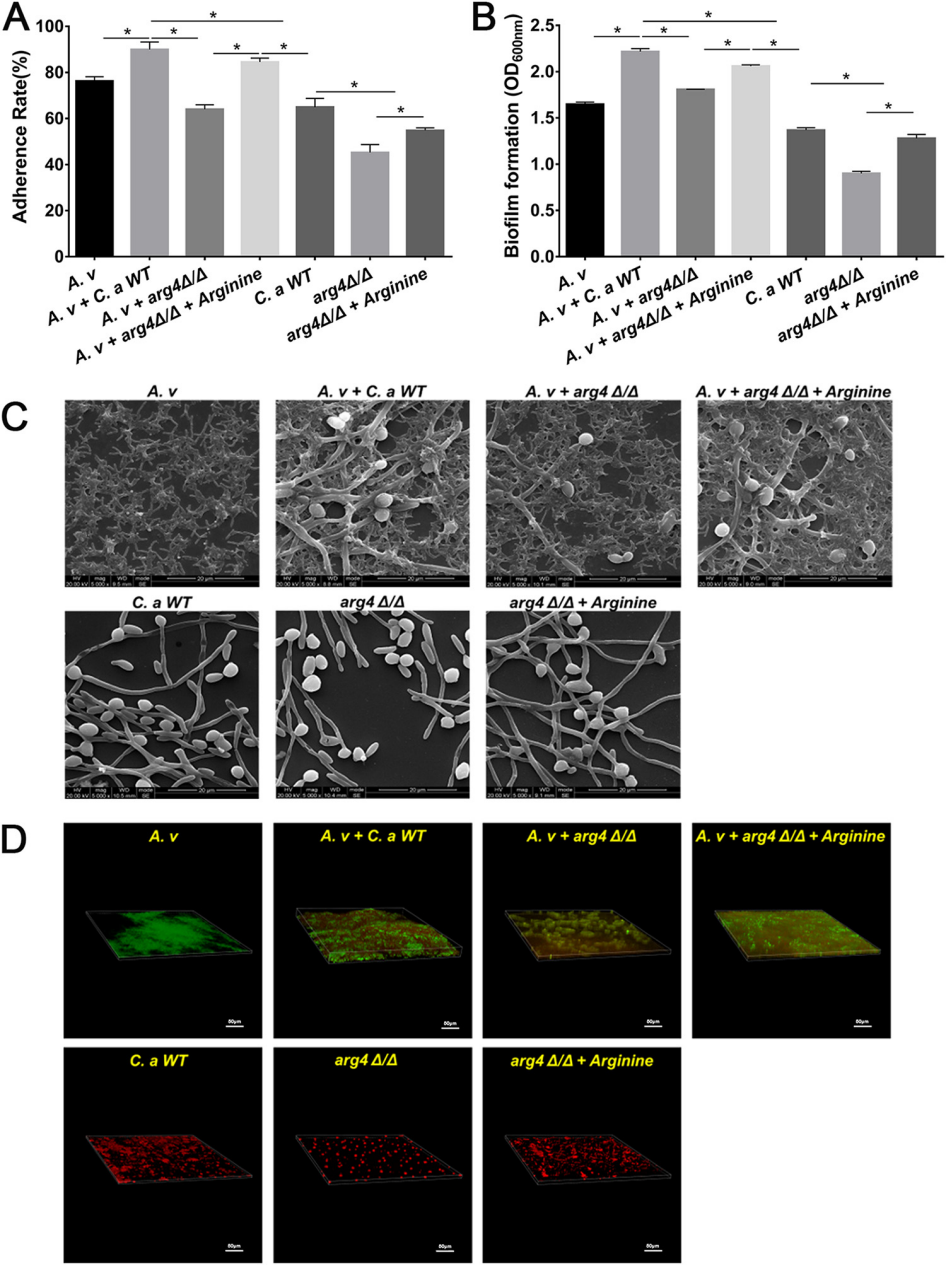
C. albicans arginine biosynthesis pathway regulated cross-kingdom interactions in dual-species biofilms. (A, B) Determination of the effects of the *arg4Δ/Δ* mutant and arginine supplementation on C. albicans growth through adherence rate (A) and biofilm formation (B) (*, *P* < 0.05). (C, D) The biofilm structures of the *arg4Δ/Δ* mutant and with arginine supplementation determined via SEM (C) and FISH observations (D). C. albicans or *arg4Δ/Δ* mutant was stained with red color while A. viscosus was stained with green color.

To confirm the essential role of the C. albicans arginine biosynthesis pathway in the regulation of its cross-kingdom interactions with A. viscosus, the *ARG4* null mutant (*arg4Δ/Δ*) was employed (Table S1). The *arg4Δ/Δ* mutant failed to exhibit enhanced cell adhesion in both the dual-species and single-species groups compared with that of the wild-type (WT) strain, while the addition of arginine recovered the promotion (Fig. 5A). The *arg4Δ/Δ* mutant exhibited reduced biofilm formation in both the dual-species and single-species biofilms compared to that of the WT strain, and the addition of arginine also reversed the reduction (Fig. 5B to D). In C. albicans WT and A. viscosus dual-species biofilm, the colonization of both C. albicans and A. viscosus were increased, compared with that in the single-species biofilm (Fig. 5D). However, the colonization of C. albicans in the *arg4Δ/Δ* and A. viscosus dual-species biofilm was not obviously increased compared with that in the *arg4Δ/Δ* single-biofilm (Fig. 5D). The addition of arginine restored the C. albicans colonization in the dual-species biofilm (Fig. 5D). These results indicated the essential roles of the arginine biosynthesis pathway of C. albicans in its cross-kingdom interactions with A. viscosus and suggested that targeting the arginine biosynthesis pathway could block their interaction in dual-species biofilm.

### The arginine biosynthesis pathway of C. albicans promoted root caries.

**FIG 6:**
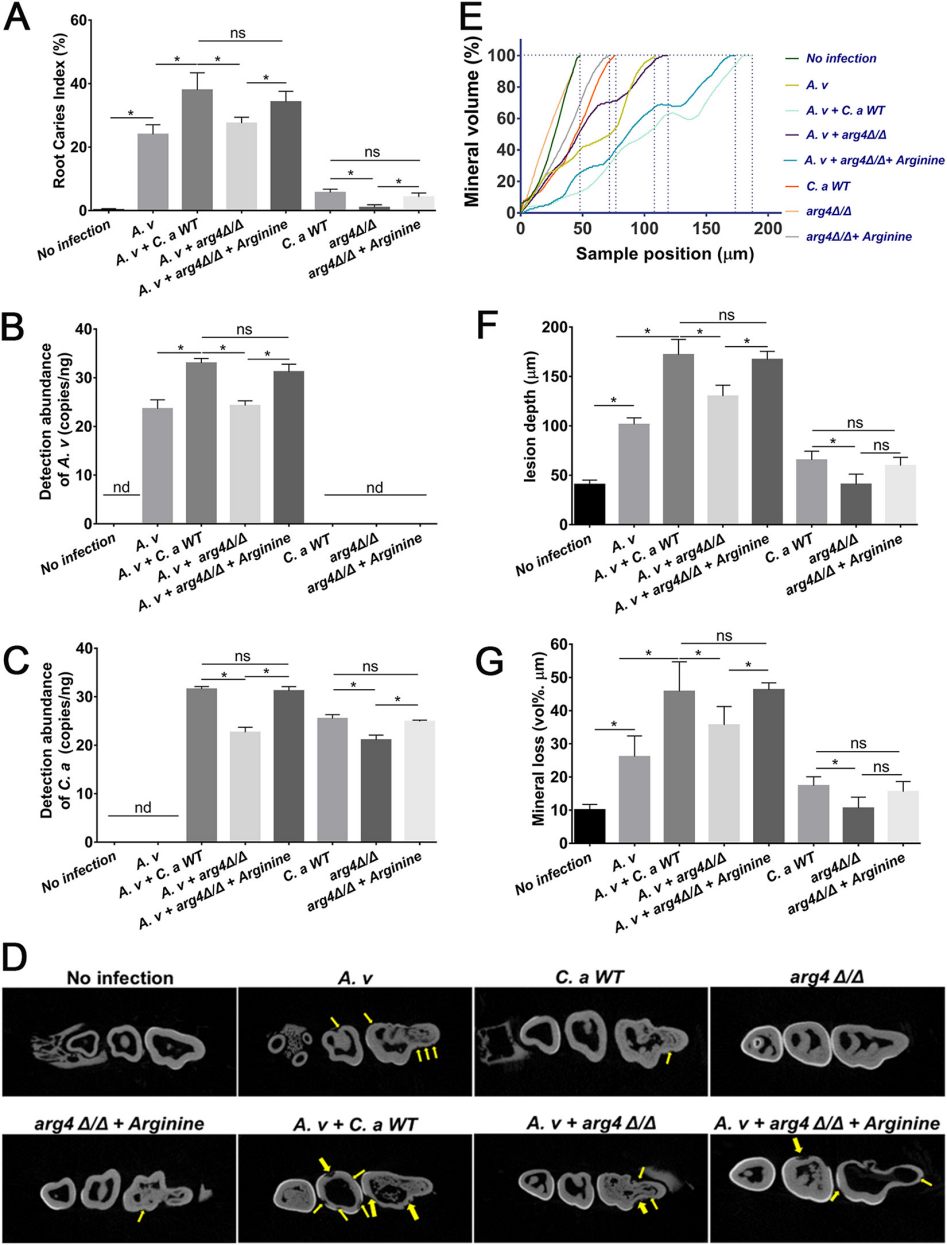
C. albicans arginine biosynthesis pathway regulated the development of root caries and affected the demineralization of the teeth in root caries. (A) Root caries index scores according to Doff’s system (*, *P* < 0.05; ns, not significant). (B, C) Levels of C. albicans (B) and A. viscosus (C) from the root-caries rats quantified via qPCR (*, *P* < 0.05; ns, not significant; nd, not detectable). (D) Representative micro-CT images of jaws from uninfected rats and rats infected with A. viscosus, A. viscosus + C. albicans, A. viscosus+ *arg4 Δ/Δ*, A. viscosus+ *arg4 Δ/Δ* + Arginine, C. albicans, *arg4 Δ/Δ*, or *arg4 Δ/Δ* + Arginine, respectively. Yellow arrows indicated root carious lesions. (E to G) The mineral volume curves (E), lesion depths (F), and mineral losses (G) of teeth from uninfected rats and rats infected with A. viscosus, A. viscosus+ C. albicans, A. viscosus+ *arg4 Δ/Δ*, A. viscosus+ *arg4 Δ/Δ* + Arginine, C. albicans/*arg4 Δ/Δ*, or *arg4 Δ/Δ* + Arginine, respectively (*, *P* < 0.05; ns, not significant).

We then investigated the contribution of the arginine biosynthesis pathway of C. albicans to the development of root caries *in vivo*. In the rat root caries model, coinfection with C. albicans WT and A. viscosus caused the most remarkable root caries lesions (Fig. 6A, D; *P* < 0.05), with elevated colonization of C. albicans and A. viscosus on the root surfaces (Fig. 6B, C; *P* < 0.05). A reduced colonization of *arg4Δ/Δ* was observed in the teeth (Fig. 6C; *P* < 0.05). Combination of *arg4Δ/Δ* and *A. viscosus* also failed to promote the colonization of A. viscosus (Fig. 6B; *P* < 0.05) and the development of root caries (Fig. 6A, D; *P* < 0.05), as the rats showed similar hard-tissue destruction, mineral volume, lesion depth and mineral loss compared to those of the rats infected by the A. viscosus single-species (Fig. 6D, G; *P* < 0.05). However, the addition of arginine increased the colonization of *arg4Δ/Δ* and A. viscosus in their combination (Fig. 6B, C; *P* < 0.05). The addition of arginine enhanced the development of root caries in rats coinfected with *arg4Δ/Δ* and A. viscosus (Fig. 6A, D; *P* < 0.05), and increased hard-tissue destruction, lesion depth and mineral loss and decreased mineral volumes (Fig. 6D, G; *P* < 0.05), indicating that the C. albicans arginine biosynthesis pathway is essential for the development of root caries.

## DISCUSSION

The incidence of root caries has increased in recent years and it has become a major oral problem in the elderly population, but the treatment of root caries is challenging (2, 31). The prevention and treatment of root caries has become one of the major issues to improve oral health. C. albicans and A. viscosus are two resident symbiotic opportunistic microorganisms in the oral cavity (32, 33), and their cross-kingdom interactions play important roles in the development of root caries. We found that the isolation frequencies and abundances of both species were significantly higher in the root caries plaque samples than in the sound root surface plaque samples (Fig. 1A to D). The abundances of the two species showed a positive correlation (Fig. 1E).

The hyphal state of C. albicans is the main virulent form, and it can efficiently mediate microbial adhesion and biofilm formation process. In the dual-species biofilm, we found that A. viscosus could promote the hyphal formation of C. albicans, thus promoting its virulence. Morse et al. (34) suggested that the coculture of C. albicans and A. viscosus could significantly increase the hyphal content in the biofilm, and the expression levels of C. albicans virulence genes, such as *ALS3*, *EPA1*, *PLD1*, *SAP4*, and *SAP6*, in the coculture biofilm were significantly increased. Our previous work also showed that cariogenic virulence such as biofilm proliferation, acid production, acid resistance, sugar production, and biofilm formation were significantly enhanced under C. albicans and A. viscosus coculture conditions (30). However, the specific pathway by which C. albicans regulates the interactions between C. albicans and A. viscosus is still unclear. In our study, KEGG enrichment analysis of the RNA-Seq-identified DEGs suggested that the C. albicans arginine biosynthesis pathway, including six upregulated genes (*ARG1*, *ECM42*, *ARG3*, *ARG4*, *ARG5*,*6*, *ARG8*) and one downregulated gene (*CAR1*), was remarkably changed in the dual-species biofilm and clinical root caries plaques (Fig. 3; Fig. S3). KEGG analysis also indicated that the alanine, aspartate, and glutamate metabolism pathway, and pantothenate and CoA biosynthesis pathway were upregulated in the dual-species group (Fig. 3). Glutamate is the essential substrate of arginine biosynthesis, while Acetyl-CoA is one of the important enzymes that metabolize glutamate to produce CoA. The produced glutamate and COA then activate the arginine biosynthesis procedure (35). KEGG analysis also showed that the lysine biosynthesis pathway was significantly enriched, and the expression levels of genes related to lysine biosynthesis (such as *HOM1*, *LYR22*, *LYR4*, etc.) were significantly upregulated in the dual-species biofilm, while an increase in intracellular lysine can promote arginine secretion (36). These upregulation pathways in the dual-species biofilms indicated that A. viscosus might enhance glutamate metabolism and CoA biosynthesis of C. albicans and then effectively promote arginine biosynthesis. In addition, A. viscosus might also increase the secretion of synthesized arginine through the upregulation of lysine biosynthesis of C. albicans. The mechanism of amino acid biosynthesis and metabolism in regulating the interaction between different species is complex. The cross talk between the different amino acid biosynthesis and metabolism from C. albicans and A. viscosus interactions require further evaluation.

In this study, we evaluated the important role of the arginine biosynthesis pathway of C. albicans in its cross-kingdom interaction with A. viscosus. The arginine biosynthesis process in the mitochondria and cytoplasm was summarized in Fig. S4A (35, 37). The deletion of *ARG4*, the key gene from the arginine biosynthesis pathway, eliminated synergistic interactions with A. viscosus, while the addition of arginine complemented the virulence deficiencies of the *arg4Δ/Δ* mutant in both the single- and dual-species groups (Fig. 5). The addition of arginine could also promote the biofilm formation of C. albicans, A. viscosus, dual-species, and hyphal formation of C. albicans (Fig. 4 and 5), while the neutral amino acids tyrosine and the acidic amino acid glutamate could not promote the biofilm formation of C. albicans and A. viscosus (unpublished data), indicating the critical role of the arginine biosynthesis pathway of C. albicans in its cross-kingdom interaction with A. viscosus (Fig. S4B), and targeting this pathway is a practical strategy to reduce the development of root caries. Further investigations are still needed to reveal the mechanisms by which the arginine biosynthesis pathway regulates the growth and virulence of A. viscosus.

Arginine is one of the most versatile amino acids in eukaryotic cells and contributes to protein synthesis, cell growth, sexual reproduction, hormone metabolism, signal transduction, osmotic pressure homeostasis, metabolic energy production, nitrogen metabolism, and urea biosynthesis (38, 39). Arginine at the appropriate concentration was essential for the growth and pathogenicity of various microorganisms. Novick et al. (40) isolated an arginine auxotroph Escherichia coli mutant and found that it grew slowly in the absence of arginine but grew at a normal rate in the presence of arginine. Hartmann et al. (41) found that arginine could help *Halobacteria* grow in the anaerobic state. Tonon et al. observed that arginine increased the growth of wine lactic acid bacteria (42). Senouci-Rezkallah et al. suggested that arginine stimulated the growth of Bacillus cereus under low-acid conditions (43). Vrancken et al. found that arginine enhanced the resistance of Lactobacillus fermentum to environmental stresses, such as acid, temperature, salt stress, and osmotic pressure factors (44). Huang’s results showed that arginine stimulated the growth of Streptococcus thermophilus *T1C2* by enhancing resistance to a low intracellular pH under high extracellular osmotic pressure (45). Zhang et al. (38) indicated that three *ARG* genes involved in arginine biosynthesis were essential for growth, conidiogenesis, sexual reproduction, hyphal growth, and pathogenicity in Magnaporthe oryzae. The M. oryzae *argΔ/Δ* mutants exhibited significantly delayed conidial germination and decreased pathogenicity, while exogenous arginine could partially restore the infection defects in invasive hyphal growth and pathogenicity (38). Similarly, the addition of arginine could restore the pathogenicity of Fusarium oxysporum *f.* sp. *melonis* arginine auxotroph mutants (46). Our results suggested that the C. albicans arginine biosynthesis pathway was significantly activated in the dual-species interactions, while hyphal and biofilm formation were also increased with the significant upregulation of hyphae-associated genes under anaerobic conditions, including *RAS1*, *NCE103*, *GPR1*, *PDE2*, *TPK1*, *UME6*, *MEP2*, *NTH1*, *TOP1*, *PTP3*, etc. (unpublished data). Arginine biosynthesis was reported to be associated with oxidative stress (47), while the transition of C. albicans yeast to hyphae also generated ROS as a by-product of oxidative phosphorylation in mitochondria (47, 48). C. albicans can also eliminate oxidative stress through the antioxidant pathway and enzymes (49–51). Our transcriptome analysis indicated that a series of antioxidant genes were significantly upregulated in the dual-species group, including *HSP78*, *HSP21*, *CIP1*, *SOD2*, *SOD5*, *FRE9*, *FRE10*, *CFL1*, *CFL2*, *CFL4*, *CFL11*, *CTR2*, *ZRT1*, and *ZRT2*, etc., indicating the increased capacity of C. albicans to eliminate oxidative stress. In addition, the low concentration of additional of arginine can directly enhance the growth, biofilm formation, and cariogenic capability of A. viscosus, indicating that the upregulated arginine was the key factor in promoting the cross-kingdom interaction. However, the specific mechanisms by which arginine regulates the effects on A. viscosus and hyphal growth of C. albicans in dual-species combination still need further investigation.

Currently, the relevance of arginine in the oral cavity is still unclear. Arginine could be catabolized by local arginase secreted from host or bacterial cells, such as macrophages and Porphyromonas gingivalis, to produce urea and ornithine, thus, increasing the production of polyamines and promote the growth of some bacteria to aggravate the inflammation and tissue destruction (52). Many studies have shown that the arginase activity was positively related to the degree of periodontal inflammation (53–55), indicating the importance of arginine in the development of oral diseases. It is worth noting that arginine could also be an effective therapeutic agent against caries, especially when combined with high-concentration fluoride (56–61), by inhibiting the growth of some cariogenic bacteria, such as Streptococcus mutans and Streptococcus sobrinus (57), and promoting the remineralization of enamel (60). However, the arginine concentrations used in these studies were high (1.5% mass fraction, or even higher at 8% to 10% mass fraction) (56–61). In our study, arginine was added at a concentration of 0.2%, which was much lower than the potential anti-caries concentrations. The concentration from our study was similar to those that promoted the growth of different microorganisms. Zhang et al. (38) indicated that exogenous arginine supplementation could partially recover the aerial hyphal growth and pathogenicity of M. oryzae *arg Δ/Δ* mutants, but the recovery effect was dependent on the concentration of arginine: the concentration with better recovery effect was 2.5 mM (approximately 0.05% mass fraction), while higher concentration led to the decreased recovery effect. Huang et al. (45) also found that 1.2g/L (approximately 0.12% mass fraction) arginine exhibited the best promotion effect on S. thermophilus *T1C2* growth and that growth was inhibited when the initial arginine concentration exceeded 1.2g/L (approximately 0.12% mass fraction). Similar results were observed by Mira et al. in Oenococcus oeni (62). Arginine inhibited S. mutans and *S. sobrinus* growth only when the concentration was over 0.4%, while arginine at a concentration ≤0.4% did not affect the growth of S. mutans or *S. sobrinus* (57). Our results indicated that arginine at low concentration could enhance the pathogenicity of A. viscosus and C. albicans, and in the root caries caused by C. albicans and A. viscosus coinfection. Therefore, the recommendation of arginine-containing caries prevention products and the arginine concentration are worth further careful consideration.

In summary, we identified for the first time that the arginine biosynthesis pathway of C. albicans was critical for the regulation of its cross-kingdom interactions with A. viscosus and for promoting the occurrence and development of root caries, while targeting this pathway can be a new practical strategy to reduce root caries.

## MATERIALS AND METHODS

### Strains and culture conditions.

A. viscosus ATCC 19246 and C. albicans WT (SC 5314, ATCC MYA-2876) were obtained from the State Key Laboratory of Oral Diseases. The *ARG4* null mutant of C. albicans was also employed to confirm the role of the arginine biosynthesis pathway (Table S1). Briefly, C. albicans BWP17 was knocked out in three genes, including *ARG4*/*URA3*/*HIS1*. Therefore, as a compromise for the deletion of *URA3* and *HIS1* genes in BWP17 and to obtain the null mutant of *ARG4* (C. albicans *arg4Δ/Δ*), BWP17 was cultured in media with additional uracil and histidine as described previously (63). A. viscosus was grown on BHY medium (brain heat infusion medium containing 5 g/l yeast extract) anaerobically (85% N_2_, 10% H_2_, and 5% CO_2_) at 37°C (30) and C. albicans WT was grown on yeast extract peptone dextrose (YPD) medium aerobically at 37°C. The coculture mixture (OD_600nm_ of each microorganism = 0.1) was grown on yeast nitrogen base (YNB) supplemented with Na_2_HPO_4_-NaH_2_PO_4_, N-acetylglucosamine, casamino acids, and sucrose medium anaerobically (85% N_2_, 10% H_2_, and 5% CO_2_) at 37°C (30). The medium of C. albicans *arg4Δ/Δ* (BWP 17) was supplemented with 0.2% histidine and 0.2% uracil.

### Microbial detection in clinical samples.

In total, 76 volunteers were recruited in our study. Thirty-nine subjects (aged 45 to 75 years old) were clinically diagnosed with root caries by radiography and clinical probing and were divided into RC group, while the other 37 caries-free subjects (aged 45 to 75 years old) acted as the HC group. All participants were in good general health. Ethical approval for the study was granted by the Institutional Review Board of the West China Hospital of Stomatology, Sichuan University (WCHSIRB-D-2020-072). Written informed consent was obtained from each participant that was recruited in this research. The sampling standards were designed as described previously (12). Briefly, in the RC group, after drying and isolating the tooth with sterile cotton rolls, the decayed plaque of root caries was collected with a dental spoon excavator. In the HC group, after drying and isolating the chosen sampling tooth, the supragingival plaque on the root surface was collected with a dental spoon excavator. Each sample was suspended in 1 mL TE buffer and stored at −80°C.

Total DNA of each sample was extracted with the DNeasy PowerSoil Kit (Qiagen, Valencia, CA, USA). Concentration and quality (A260 nm and A280 nm) measurements of the extracted DNA were performed with a NanoDrop ND-1000 spectrophotometer (Thermo Fisher Scientific, Waltham, MA, USA).

The detection rates of C. albicans and A. viscosus were determined by PCR (12). The detection abundances of C. albicans and A. viscosus were quantified by qPCR (12, 64) according to the standard curves of A. viscosus and C. albicans (65). Correlation analysis and linear regression models were constructed to observe the change trends of the abundances of the two microorganisms. The primers used in this part were listed in Tables S2 and S3.

### Adherence assay.

The adhesion assay was performed as described previously (66). Biofilms were formed in 48-well plates (single species groups: 500 μL C. albicans, *arg4Δ/Δ* or A. viscosus, respectively, for each well; dual species groups: 250 μL C. albicans or *arg4Δ/Δ* + 250 μL A. viscosus for each well) under stationary conditions after 24-h incubation in YNBB medium anaerobically (85% N_2_, 10% H_2_, and 5% CO_2_) at 37°C. The total cells including the cells from the suspension and the cells that were adhered to the well bottom were thoroughly mixed, and then OD_600nm_ was recorded to quantify the total bacteria (OD_600nm_ of total cells). Adherent cells were obtained by removing the suspension and resuspending the remaining cells in equal volume of medium (OD_600nm_ of adherent cells). The adhesion rate was calculated with the formula: adhesion rate = OD_600nm_ of adherent cells/OD_600nm_ of total cells (including the adherent cells and the suspension cells) × 100%.

### Crystal violet assay and CFU counts.

In 96-well plates, 24-h biofilms were produced (single species groups: 200 μL C. albicans, *arg4Δ/Δ* or 200 μL A. viscosus, respectively, for each well; dual species groups: 100 μL C. albicans/*arg4Δ/Δ* + 100 μL A. viscosus for each well) under stationary conditions in YNBB medium anaerobically (85% N_2_, 10% H_2_, and 5% CO_2_) at 37°C. The total biomass of each biofilm was quantified by crystal violet assay as previously described (30). The biofilms were sequentially fixed with methanol and stained with 0.1% (wt/vol) crystal violet for 15 to 30 min. The suspension was removed, and the cells were resolubilized with 33% (vol/vol) glacial acetic acid. Total biomass was determined with OD_600nm_ of the suspension. To quantify the viable cells in biofilms, CFU counts were performed as described previously (30). Briefly, the biofilms were scraped off and resuspended with equal volume of medium. Then, the suspensions were 1:10 serially diluted and viable biofilm cells were quantified by CFU counts after plating the proper dilutions on YPD agar and incubating for 24 h.

### Scanning electron microscopy observation and fluorescence *in situ* hybridization observation.

To observe the biofilm structure, scanning electron microscopy (SEM) observation and fluorescence *in situ* hybridization (FISH) observations were performed. Then, 24-h biofilm samples were produced on sterile glass slides at the bottom of each well of 24-well plates (single species groups: 1,000 μL C. albicans, *arg4Δ/Δ* or A. viscosus, respectively, for each well; dual species groups: 500 μL C. albicans/*arg4Δ/Δ* + 500 μL A. viscosus for each well) under stationary conditions in YNBB medium anaerobically (85% N_2_, 10% H_2_, and 5% CO_2_) at 37°C. SEM analysis was carried out as previously described (30). Each sample was observed by SEM imaging (FEI, Hillsboro, USA) at 5,000× magnification. The FISH procedure was performed as previously described (30) and observed with an Eclipse FV1000 inverted confocal laser scanning microscope (Olympus Corporation, Japan). The sequences of oligonucleotide probes (30) were listed in Table S4. The probes were synthesized by Sangon Biotech (Shanghai, China).

### Anthrone-sulfuric acid assay.

The water-insoluble EPS production ability of 24-h biofilms were analyzed by anthrone-sulfuric acid assay. In 96-well plates, 24-h biofilms were produced (single species groups: 200 μL C. albicans, *arg4Δ/Δ* or A. viscosus, respectively, for each well; dual species groups: 100 μL C. albicans/*arg4Δ/Δ* + 100 μL A. viscosus for each well) under stationary conditions in YNBB medium anaerobically (85% N_2_, 10% H_2_, and 5% CO_2_) at 37°C. The biofilms were resuspended, and the precipitates were obtained and washed with sterile water to remove the water-soluble EPS. Then, each water-insoluble EPS sample was extracted with 0.4M NaOH under agitation for 2 h. Three milliliters of 0.2% anthrone-sulfuric acid reagent was mixed into each supernatant sample and then incubated in a water bath at 95°C for 6 min. The water-soluble EPS production ability was determined by OD_625nm_.

### RNA sequencing and data analysis.

Total RNA in each sample was extracted with TRIzol reagent (Invitrogen, Carlsbad, USA). RNA sequencing (RNA-Seq) was performed by Illumina NovaSeq (Shanghai Personal Biotechnology Co., Ltd., China) as described elsewhere (67).

A total of 6,030 genes were analyzed. Differential gene expression analysis was performed using fragments per kilobase per million (FPKM) values. The Pearson correlation coefficient was estimated to analyze the correlation of gene expression levels between samples and principal-component analysis (PCA) was used to cluster samples in each group. Differentially expressed genes (DEGs) were defined with the criteria of absolute log_2_-fold change (FC) > 1 and adjusted *P* value < 0.05. DEGs were regarded as upregulated if their expression levels in dual-species biofilm samples were higher than those in the Candida albicans single-species biofilm, and vice versa. The expression of DEGs in each treatment was visualized as a volcano plot and heatmap. DEGs were submitted for functional enrichment analyses to Gene Orthology (GO) and KEGG annotations.

### Analysis of the gene expression levels in biofilms.

The biofilms were collected and quantitative PCR (qPCR) was performed to evaluate the expression levels of arginine biosynthesis-associated DEGs (*ARG1*, *ARG3*, *ARG4*, etc.). RNA isolation with TRIzol Reagent (Invitrogen, Carlsbad, CA, USA) and purification procedures were conducted as previously described (68). To synthesize first-strand cDNAs, RNA reverse transcription was performed with a PrimeScript RT reagent kit with gDNA Eraser (TaKaRa Biotechnology, Japan). Specific primers for the tested genes were designed using Primer3Plus (http://www.primer3plus.com/cgi-bin/dev/primer3plus.cgi) and were listed in Table S5. The qPCR mixture and procedure were carried out as previously described (68). Relative fold changes in the expression of associated genes were evaluated with the 2^-ΔΔCt^ method (69), and the 18S rRNA gene expression level was used to normalize the expression level of different genes.

### Gene expression analysis in clinical samples.

In total, another 20 volunteers were recruited. Ten subjects (aged 45 to 75 years) were clinically diagnosed with root caries by radiography and clinical probing and were divided into the RC group, while the other 10 caries-free subjects (aged 45 to 75 years) acted as the HC group. All participants were in good general health. Ethical approval for the study was granted by the Institutional Review Board of the West China Hospital of Stomatology, Sichuan University (WCHSIRB-D-2020-072). Clinical plaque samples were collected as described above. RNA from each sample was extracted with TRIzol Reagent (Invitrogen, Carlsbad, CA, USA) and qPCR was performed to compare the expression levels of genes in the arginine biosynthesis pathway in the RC group and HC group.

### Root caries rat model.

The rat model was established to investigate the promotion ability of C. albicans and A. viscosus interactions on root caries and the corresponding pathogenesis *in vivo*. The experiment was started after approval was obtained from the animal research committee of West China School of Stomatology, Sichuan University (WCHSIRB-D-2020-127). Male 17-day-old specific pathogen free (SPF) Sprague-Dawley (SD) rats purchased from Dashuo Inc. (Chengdu, China) were used for the *in vivo* experiment (five rats in each group). The root caries model was established as described in a previous study (12). Briefly, the rats were fed with 5% (wt/vol) sucrose-containing water and caries-promoting diet (Diet 2000) every day. The rats were infected daily for 3 consecutive days with A. viscosus, C. albicans WT, *arg4Δ/Δ* mutant, and C. albicans-*A.viscosus* combinations according to the designated groups (10^9^ CFU/mL, 200 mL each rat). Ten days after the initial infection, the rats were anesthetized and underwent the gingivectomy surgery. On days 38 to 40, the rats were reinoculated with microbes. On day 66, the rats were sacrificed and the jaws were removed aseptically. The dental plaque of each jaw was collected to detect the abundances of microorganisms through qPCR as described above. Each jaw was stained with mercurochrome for 18 h to record the root caries score according to Doff’s criterion (70). Then, the jaws were subjected to the micro–computed tomography (μCT 50, SCANCO Medical AG, Brüttisellen, Switzerland) analysis (71). They were scanned at a medium resolution, with parameters of 70 kVp and 200 μA. Each sample was rotated 360° within 14.3 min. SCANCO evaluation software version 1.1.11.0 (SCANCO Medical AG) was used to acquire and analyze Micro-CT images. A line in the selected sectional view of each jaw was chosen as the region of interest (ROI) to be quantitatively analyzed. The mineral volume, lesion depth, and mineral loss of the ROI were measured by SCANCO evaluation software to evaluate the degree of root caries.

### Statistical analysis.

For the clinical sample detections, differences between the two groups were compared with *t* test or Kruskal-Wallis analysis after a homogeneity test of variance with Levene’s test. For the other experiments, differences among multiple groups were compared using one-way ANOVA and *post hoc* Tukey’s multiple comparisons after a homogeneity test of variance with Levene’s test, and two independent groups were analyzed with *t* test after the homogeneity of variance test. Statistical analysis was performed using SPSS software (Version 20.0; IBM Corp., Armonk, USA) with a significance level of 0.05, and then all figures were generated with GraphPad Prism7 software (version 7.00 for Windows; GraphPad Prism, Inc, La Jolla, USA).

### Data availability.

RNA sequencing data have been deposited in the public database Sequence Read Archive with accession no. (PRJNA753272). All data sets generated and/or analyzed in the current study are available from the corresponding author on reasonable request.
