# Supplementary material for: The Arginine Biosynthesis Pathway of Candida albicans Regulates Its Cross-Kingdom Interaction with Actinomyces viscosus to Promote Root Caries
Source: Microbiol Spectr. 2022 Jul 13;10(4):e00782-22. doi: 10.1128/spectrum.00782-22 (PMC9430244; doi:10.1128/spectrum.00782-22)
Supplement: Supplemental file 1 — Fig. S1 to S4; Tables S1 to S5. Download spectrum.00782-22-s0001.pdf, PDF file, 0.4 MB [file spectrum.00782-22-s0001.pdf]

The arginine biosynthesis pathway of *Candida albicans* regulates its cross-kingdom interaction with *Actinomyces viscosus* to promote root caries

Running Title: *C. albicans* and *A. viscosus* promote root caries

Authors: Kaixin Xiong[1], Hualing Zhu[1], Yanyao Li[1], Mengzhen Ji[1], Yujia Yan[1], Xuan Chen[1], Yaqi Chi[1], Xueqin Yang[1], Ling Deng[1], Xuedong Zhou[1], Ling Zou[2,*], Biao Ren[1,*]

[1] State Key Laboratory of Oral Diseases, National Clinical Research Center for Oral Diseases, West China School of Stomatology, Sichuan University, Chengdu 610041, China

[2] State Key Laboratory of Oral Diseases, National Clinical Research Center for Oral Diseases, Department of Conservation Dentistry and Endodontics, West China School of Stomatology, Sichuan University, Chengdu, 610041, China

***Corresponding Author:**

Dr. Ling Zou, Associate Professor, State Key Laboratory of Oral Diseases, National Clinical Research Center for Oral Diseases, Department of Conservation Dentistry and Endodontics, West China School of Stomatology, Sichuan University, Chengdu 610041, China (Email: zouling@scu.edu.cn).

Dr. Biao Ren, Associate Professor, State Key Laboratory of Oral Diseases, National Clinical Research Center for Oral Diseases, West China School of Stomatology, Sichuan University, Chengdu 610041, China (Email: renbiao@scu.edu.cn).

Supplementary Figure S1

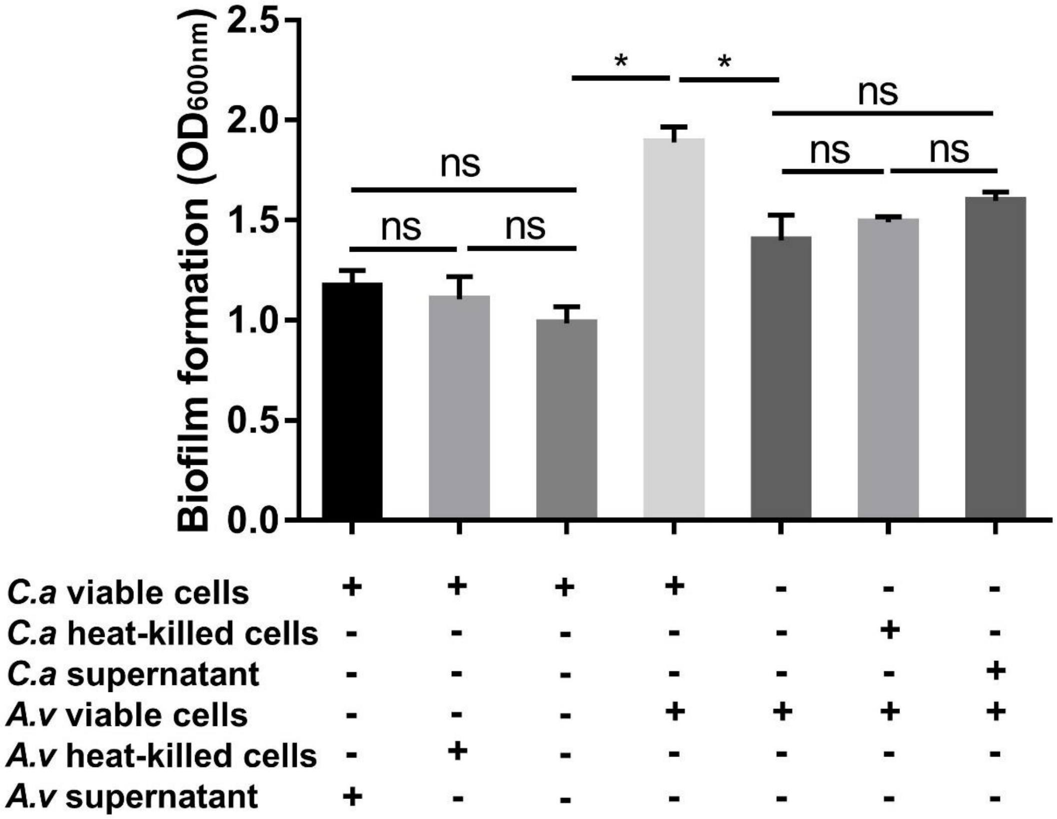

Supplementary Figure S1: The interactions from the combinations of viable cells, heat-killed cells and cell supernatants of *C. albicans* and *A. viscosus*, respectively. The biofilm formation in each group was calculated by Crystal Violet assay. (*, $p < 0.05$; ns, not significant)

Supplementary Figure S2

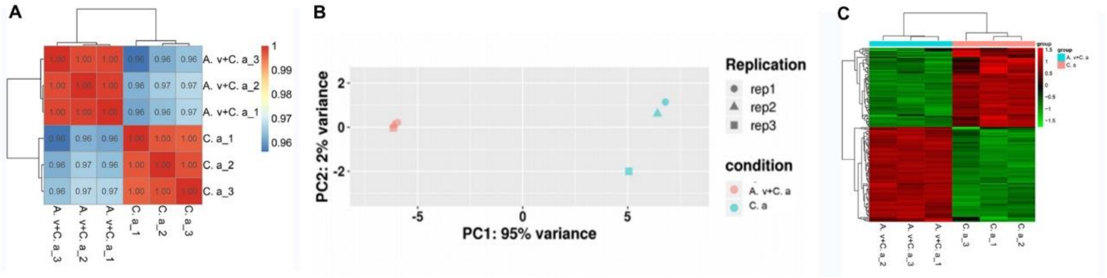

Supplementary Figure S2: RNA-Seq analysis of *C. albicans* single biofilm and *A. viscosus* + *C. albicans* dual-species biofilm

(A, B): Correlation analysis of patterns of gene expression and Principal component analysis (PCA) of FPKM profiles in *C. albicans* single species group and *C. albicans* and *A. viscosus* dual-species group;

(C): Heatmap of centered and scaled FPKM values of DEGs.

Supplementary Figure S3

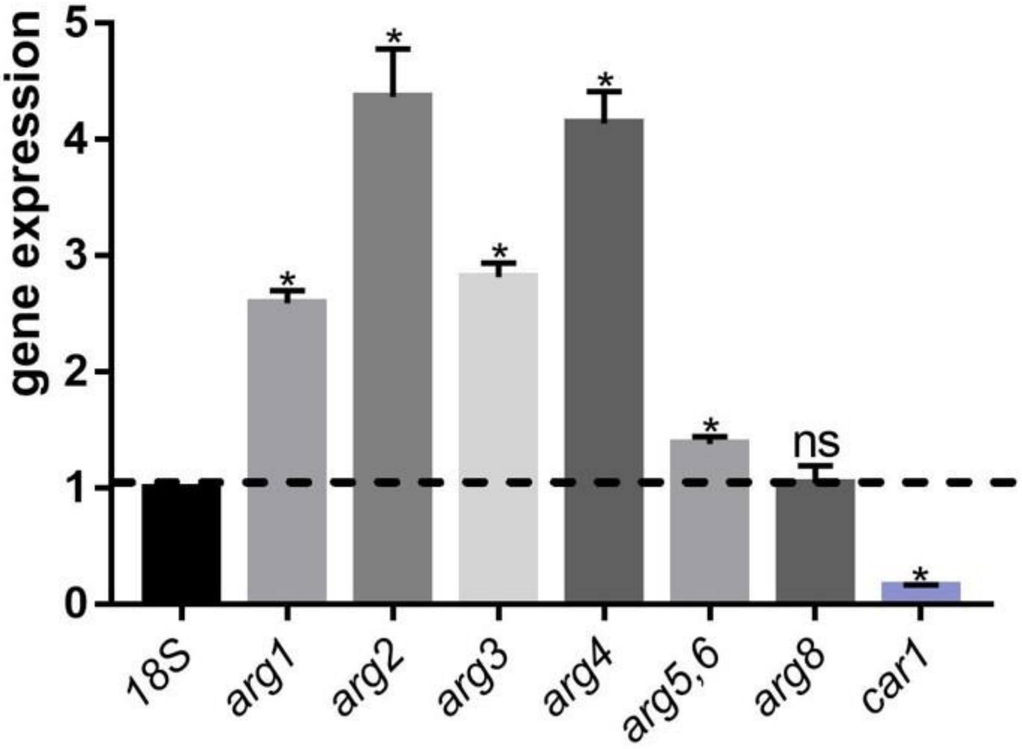

Supplementary Figure S3: Expression levels of genes in arginine biosynthesis pathway. The arginine-associated genes expression of *C. albicans* in the *C. albicans* + *A. viscosus* dual-species biofilm were quantified by qPCR. The control group was *C. albicans* single species biofilm. (*, $p < 0.05$; ns, not significant)

Supplementary Figure S4

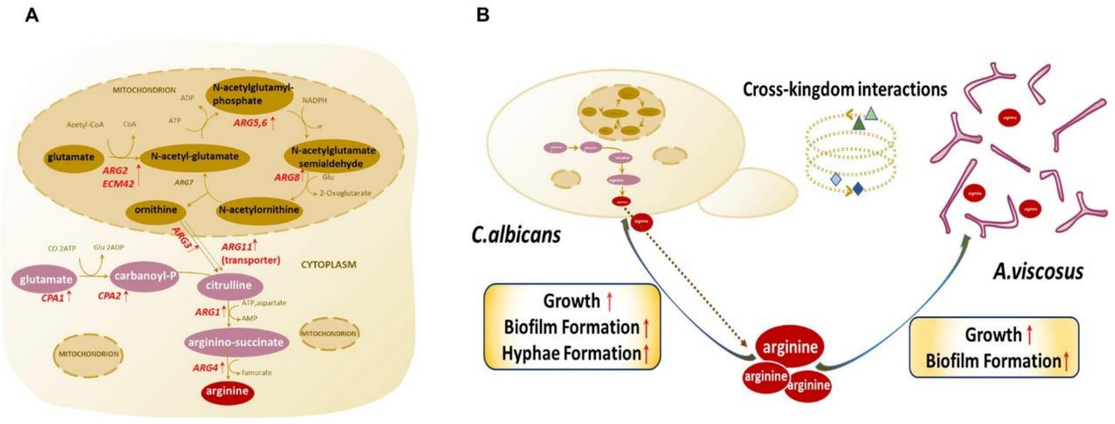

Supplementary Figure S4: *C. albicans* arginine biosynthesis pathway and its role in the cross-kingdom interactions with *A. viscosus*

(A): Representation of the arginine biosynthetic pathway in the mitochondria and cytoplasm of yeast cells. Expressions of the genes highlighted in red were increased in *C. albicans* + *A. viscosus* dual-species biofilm compared to those in *C. albicans* single-species biofilm. The gene shown in black (*ARG7*) had no detectable change in expression. CoA, coenzyme A.

(B): The cross-kingdom interactions between *C. albicans* and *A. viscosus* mediated by the *C. albicans* arginine biosynthesis pathway.

Supplementary Table S1. *C. albicans* strains used in this work

**Strain**	**Relevant genotype**	**phenotype**
SC5314	Wild type: *URA3* /URA*3*<br>*HIS1* /HIS*1*<br>*ARG4* /ARG*4*	Wild type: Ura His Arg
BWP17	Mutant type: URA*3*Δ /URA*3*Δ<br>*HIS1*Δ /HIS*1*Δ<br>*ARG4*Δ /ARG*4*Δ	Mutant type: Ura^{-} His^{-} Arg^{-}

Supplementary Table S2. PCR primers used in this study

Species	Primers	Sequences (5'–3')	Amplicon size, bp	References
C. albicans	*C. a*-detection-F	CGATTCAGGGGAGGTAGTGAC	276 bp	(1)
	C. a-detection-R	GGTTCGCCATAAATGGCTACCAG		
A. viscosus	*A. v*-detection-F	TCTGCGATTACTAGCGACTCC	785 bp	(2)
	A. v-detection-R	TCGTAGGCGGCTGGTCGC		

Supplementary Table S3. qPCR primers used for microbe quantification in this study

Species	Primers	Sequences (5'–3')	References
C. albicans	*C. a*-F	TTTATCAACTTGTCACACCAGA	(3)
	C. a-R	ATCCCGCCTTACCACTACCG	
A. viscosus	*A. v*-F	TCTGCGATTACTAGCGACTCC	(2)
	A. v-R	TCGTAGGCGGCTGGTCGC	

Supplementary Table S4. Sequences of oligonucleotide probes used in this study

Species	Oligonucleotide probes	Probe sequences	References
C. albicans	EUK516 (labelled with Cy3)	5′-ACCAGACTTGCCCTCC-3′	(4)
A. viscosus	ACT218 (labelled with FITC)	5′-CGAGCCCCATCCCCCACCA-3′	(4)

Supplementary Table S5. Specific primers for tested genes

Genes	Primers	Sequences (5’–3’)
C. albicans-18S	*18S*-F	TCTTTCTTGATTTTGTGGGTGG
	18S-R	TCGATAGTCCCTCTAAGAAGTG
C. albicans-ARG1	*ARG1*-F	TCCAGTAGAATTATTCATTGAAGCTAA
	ARG1-R	ACAACCTCTGGATTTAATACCAAT
C. albicans-ECM42	*ECM42*-F	TTGCCAATCGATAAAATCTTAGG
	ECM42-R	GGTTGTGCAAATTGCTGTTG
C. albicans- ARG3	*ARG3*-F	CGATTGCTTGTTTGAAATTAGG
	ARG3-R	TTTGTTCCTTAGCTAATTTTTCAGC
C. albicans-ARG4	*ARG4*-F	GAACGTCGATTAGGAGAAATCA
	ARG4-R	CGAAAATTCTCATATCGGTAGCA
C. albicans-ARG5,6	*ARG5,6*-F	TGGCGTATTCACATCTGCTC
	ARG5,6-R	CTCTCGGTTTGCTTACCTTCA
C. albicans-ARG8	*ARG8*-F	TGGGATGAAAGATGCTAGTCG
	ARG8-R	CCGGATTTATTGATTTCCCATA
C. albicans-CAR1	*CAR1*-F	GCAAGTTTGGTACACGATCC
	CAR1-R	CAACCGTGTAAATTGCCTGA

References:

1. Baumgartner JC, Watts CM, Xia T. 2000. Occurrence of Candida albicans in infections of endodontic origin. J Endod 26:695-8.
2. García-García A, Coronel-Martínez J, Leon DC, Romero-Figueroa MDS, Caballero-Pantoja YE, Manzanares-Leal GL, Rodriguez-Morales M, Sandoval-Trujillo H, Ramírez-Durán N. 2017. Detection of Actinomyces spp. in cervical exudates from women with cervical intraepithelial neoplasia or cervical cancer. J Med Microbiol 66:706-712.
3. Luo G, Mitchell TG. 2002. Rapid identification of pathogenic fungi directly from cultures by using multiplex PCR. J Clin Microbiol 40:2860-5.
4. Deng L, Li W, He Y, Wu J, Ren B, Zou L. 2019. Cross-kingdom interaction of Candida albicans and Actinomyces viscosus elevated cariogenic virulence. Arch Oral Biol 100:106-112.
